# Storage Management Strategy in Mobile Phones for Photo Crowdsensing

**DOI:** 10.3390/s20082199

**Published:** 2020-04-13

**Authors:** En Wang, Zhengdao Qu, Xinyao Liang, Xiangyu Meng, Yongjian Yang, Dawei Li, Weibin Meng

**Affiliations:** 1 Department of Computer Science and Technology, Jilin University, Changchun 130012, China; wangen@jlu.edu.cn (E.W.); yyj@jlu.edu.cn (Y.Y.); 2 Department of Software, Jilin University, Changchun 130012, China; quzd2217@mails.jlu.edu.cn; 3 College of Business Administration, Anhui Finance and Economics University, Anhui 233030 China; 20181332@aufe.edu.cn; 4 Department of Computer Science, Montclair State University, Montclair, NJ 07043, USA; dawei.li@montclair.edu; 5 Department of Computer Science and Technology, Tsinghua University, Beijing 100084, China; mwb16@mails.tsinghua.edu.cn

**Keywords:** mobile crowdsensing, edge computing, photo crowdsensing, storage management, utility-based

## Abstract

In mobile crowdsensing, some users jointly finish a sensing task through the sensors equipped in their intelligent terminals. In particular, the photo crowdsensing based on Mobile Edge Computing (MEC) collects pictures for some specific targets or events and uploads them to nearby edge servers, which leads to richer data content and more efficient data storage compared with the common mobile crowdsensing; hence, it has attracted an important amount of attention recently. However, the mobile users prefer uploading the photos through Wifi APs (PoIs) rather than cellular networks. Therefore, photos stored in mobile phones are exchanged among users, in order to quickly upload them to the PoIs, which are actually the edge services. In this paper, we propose a utility-based Storage Management strategy in mobile phones for Photo Crowdsensing (SMPC), which makes a sending/deleting decision on a user’s device for either maximizing photo delivery ratio (SMPC-R) or minimizing average delay (SMPC-D). The decision is made according to the photo’s utility, which is calculated by measuring the impact of reproducing or deleting a photo on the above performance goals. We have done simulations based on the random-waypoint model and three real traces: *roma/taxi*, *epfl*, and *geolife*. The results show that, compared with other storage management strategies, SMPC-R gets the highest delivery ratio and SMPC-D achieves the lowest average delay.

## 1. Introduction

Recent years have witnessed the obvious growth of mobile devices, where all kinds of sensors (e.g., locator, camera, accelerometer) are implanted to make the mobile devices have a powerful sensing ability. For this reason, a mobility-based crowdsourcing way called *mobile crowdsensing* is presented [1] to gather a group of mobile users for collectively finishing a common sensing task using their mobile devices. Particularly, a novel case of mobile crowdsensing is collecting graphical information through the cameras of users’ mobile phones [2,3].

For example, in a disaster area or a battle field network, the control center requires the pictures of the specific targets. The mobile devices of the people around the targets could be used to take pictures and the holders could upload the photos to the control center. Another case is that Google Maps services provide customers with a visual feeling of the specific places, through the street cameras. Obviously, a picture could give the customers richer content than text description. A well-known application Waze [4,5] utilizes the thought of crowdsensing to provide the timely road traffic situation. Drivers could share the road traffic information through taking photos for the accidents, dangers, and also road conditions; then, the sensing data could be efficiently uploaded to the control center and translated into the related knowledge, which could serve back for the drivers.

**Figure 1:**
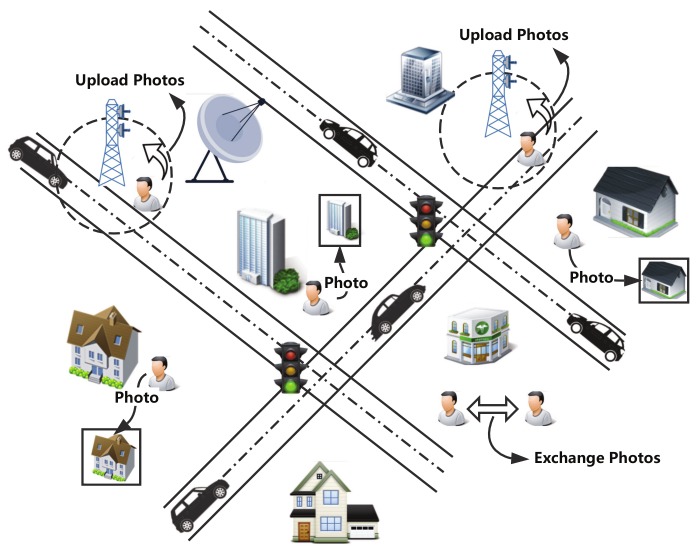
A running example of photo crowdsensing.

There are some works focusing on mobile crowdsensing including system design [6,7,8], worker recruitment strategies [9,10,11,12,13], incentive mechanisms [14,15,16,17,18,19], and so on. However, there have been a small amount of works focusing on mobile photo crowdsensing. More importantly, an important issue is the storage problem in mobile photo crowdsensing because the storage space for a phone to serve for the crowdsensing application is usually limited; even the total storage space is enough (most space is used by the other applications in user’s daily life). As shown in Figure 1, users move around an area to cooperatively perform a mobile crowdsensing task. There are some Wifi APs regarded as the PoIs, which are actually the edge services in Mobile Edge Computing (MEC) [20]. When they see the targets (buildings or sights), they take photos and store the photos in their storage. Users need to upload the photos to the server in order to finish the photo crowdsensing task. They prefer uploading the photos through PoIs freely rather than uploading through a costly cellular network [21]. Therefore, they copy the photos when they enter the communication area of each other, in order to upload the photos as soon as possible. However, each mobile phone has a limited amount of storage that serves for crowdsensing tasks. In order to efficiently finish the photo crowdsensing task, users need to decide which photo to copy first when the connection duration among users is limited. Moreover, they also need to decide which photo to delete first when the storage of the mobile phone is overflowed. Of course, the final purpose is to upload as many photos as possible; then, the crowdsensing task could be well finished.

This paper studies a storage management framework, which consists of a parameter collection module, utility calculation module, and decision module in mobile phones for photo crowdsensing. The purpose of this work is to make a smart decision about which photo should be sent or deleted. The smart decision (decision module) is based on measuring the increase or decrease for every photo copy on either the photo delivery ratio or average delivery delay. The impact is quantified as the photo’s utility (utility calculation module), which is achieved by corresponding ordinary differential equations (ODEs). The ODE solution calculates each photo’s utility value, according to the collection of the following states: the number of photo copies, and the number of users that have ever stored the photo copy (parameter collection module). Finally, we propose a utility-based Storage Management strategy in mobile phones for Photo Crowdsensing (SMPC). We have done the simulations, which prove that the proposed storage management strategy achieves a good performance.

The main contributions of this paper are briefly summarized as follows:

- We propose a storage management framework, which includes the parameter collection module, utility calculation module, and decision module to address the photo crowdsensing problem in mobile phones.
- We propose two utility-based Storage Management strategies in mobile phones for Photo Crowdsensing (SMPC), one is for maximizing delivery ratio (SMPC-R), and the other one is for minimizing average delay (SMPC-D).

The remainder sections are organized as follows: In Section 2, we describe the process to formulate the question to mathematical linguistics. The storage management framework is presented in Section 3. In Section 4, we propose two utility-based storage management strategies in mobile phones for photo crowdsensing, for the goals of delivery ratio and average deliver delay, respectively. In Section 5, we test the performance of the storage management strategies in this paper through many groups of simulations. We review the related work in Section 6 and conclude the paper in Section 7.

## 2. Related Work

There are two parts of existing research related to the work in this paper: buffer management in DTNs [22,23] and photo crowdsourcing.

### 2.1. Buffer Management in DTNs

Elwhishi et al. [24] present a new framework to schedule message, thus increasing the forwarding radio and lowering the propagation delay. Wang et al. [25] propose a strategy to dispatch or drop the message, in order to make full use of the network resource and reduce the buffer is overflowed. Krifa et al. [26] present an efficient buffer control strategy about the network using the theory of collision-based message propagation. Based on the result of [26], Krifa et al. [27] make progress and put forward a joint scheduling and drop strategy to optimize the metrics; furthermore, they propose a distributed algorithm to approximate the optimal algorithm. Moreover, Krifa et al. [28] utilize collected statistics to estimate all useful parameters, in order to enhance their previous work. Aruna et al. [29] regard the DTN routing as a resource allocation problem, then propose a DTN routing protocol to optimize significant performances such as the worst transmission delay and so on. Matzakos et al. [30] consider both the delivery probability and delay requirements in DTN and propose a distributed buffer management approach based on the heterogeneous contact rates and sparse contact graphs.

The above research focuses on the buffer-management of nodes in DTNs. The works are similar to this paper. However, the strategies proposed in DTNs could not be directly used in mobile phones for photo crowdsensing.

### 2.2. Photo Crowdsourcing

Zhou et al. [31] propose an urban traffic monitoring system by using bus riders’ mobile phones which can turn buses into probes for monitoring buses’ travel situations and inferring instant traffic map of the city. Rula et al. [32] propose an approach which leverages the built-in incentives of location-based gaming and social applications to exert limited control over the actions of participators. Siahaan et al. [33] study the influence of different experimental settings and rating scales on the reliability and repeatability of aesthetic appeal assessments in the process of collecting picture aesthetic appeal ground truth data by a systematic way. Wang et al. [34] propose a crowdsourcing based scene reporting system (RVShare). When the real-time picture of a location is requested, the RVShare system could provide the picture by means of the person who is passing by the location. Moreover, in order to perfect the system, they also design a task allocation scheme, a reward scheme, and a processing flow to deal with the pictures uploaded. Considering the security of the data such as photos, Wu et al., propose a data protection and recovery method based on the incentive [35] and a prediction-based risk defense strategy [36] for the data. Zhuo et al. [37] propose a tripartite architecture for data aggregation and analysis to mitigate the pressure of resource constrained requesters in mobile crowdsourcing by means of cloud support; they achieve privacy-preserving of data and identity of workers. The requester is able to verify the results received from the cloud. Wu et al. [38] propose a picture crowdsourcing framework through disruption tolerant networks (DTNs) as well as a picture selection algorithm, which uses the photo meta data to measure the priority of photos and takes the coverage of overlap photos into consideration. Wu et al. [39] design a pricing incentive mechanism based on quality of videos named Vbargain for the purpose of encouraging mobile users to cooperate to deliver video data effectively and easing the burden of mobile data traffic. They take the marginal gains of video quality and its duplicate into consideration and view the process of data delivery as a behavior of market to stimulate the workers. Zhou et al. [40] focus on selecting a proper photo subset of an area from the server to a requester. Based on the coverage on areas and quality on the views, they propose a photo selection approach that contains two schemes: basic and PoI number-aware photo selection schemes.

The above works focus on utilizing the photo crowdsourcing to efficiently finish the crowsensing task. However, as far as we know, there is no work focusing on storage management in mobile phones for photo crowdsensing. Above all, this paper is the first work to propose a storage management strategy in mobile phones for photo crowdsensing.

## 3. Network Model and Problem Formulation

### 3.1. Network Model

**Figure 2:**
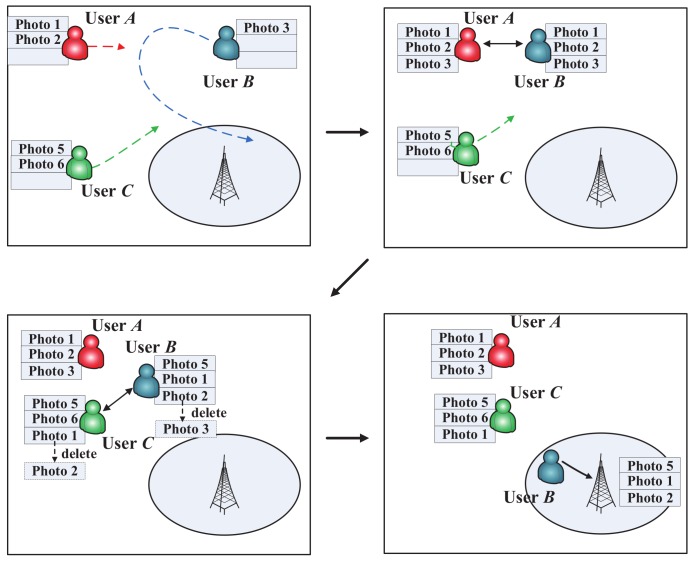
A running example of the storage management problem in mobile phones for photo crowdsensing. User *A* with photos 1 and 2 encounters user *B* with photos 3; they exchange photos in the epidemic manner. After a period of time, user *B* encounters user *C*; they exchange photos and delete the photo with the lowest utility when the storage is overflowed. Finally, when user *B* enters a PoI, it uploads all the photos successfully.

We pay attention to a mobile network for photo crowdsensing including *N* mobile users, which will randomly take photos (the requesters publish some tasks to take the photos for the specific targets) through their mobile phones. The photos need to be uploaded before a deadline. We assume that all the photos have a uniform time to live (TTL). To minimize total uploading cost of all the photos, users prefer uploading the photos through a free-Wifi connection, which is defined as a PoI, rather than uploading them costly. In all the PoIs, users could upload all the photos in the storage. Each user has a uniform communication range *d*, when a pair of users enter the communication range of each other, they could exchange some photos, whose number is decided by their contact time. A longer contact time leads to a larger number of exchanged photos. The exchange process is an epidemic [41] copy process, which utilizes all the transmission probabilities to copy the photos to all the encounters. Here, we assume that some incentive mechanisms have been designed to make users prefer assisting with finishing the MCS tasks, while the incentive work is not considered in this paper. A photo could just be deleted in the following two situations: successfully uploaded to a PoI, or deleted by the management strategy when the storage is overflowed. Before uploading to a PoI, the photos need to be stored in a mobile phone’s storage. We assume that every mobile phone has a uniform storage space, and all the photos are also the same size. When the storage is overflowed, some photos should be deleted from the storage. The network environment is shown in Figure 2. The main symbols are listed in Table 1.

### 3.2. Problem Formulation

As described in the network model, in order to finish the photo crowdsensing, users take photos when they see some interesting activities or sights. In addition, they exchange photos in the epidemic manner, in order to deliver the photo to a PoI. In this way, users cooperatively finish a phone crowdsensing task through their mobile phones. However, the epidemic exchanging strategy consumes a large number of storage resources, and users’ mobile phones also have a limited storage space, which could not be regarded as an abundant resource. Therefore, in order to achieve a good photo crowdsensing result, we should propose a storage management strategy in mobile phones.

Through the above descriptions, when a user takes a photo in its storage, we could regard the user as source and consider the PoIs as destinations. The purpose of photo crowdsensing is to deliver the photos from sources to destinations with the help of the other users. However, taking the limited storage space of mobile phones into consideration, our work mainly solves the next two problems: (1) when one or more photos are stored in a user’s phone storage and the user could not decide whether the connection could be enough to copy these photos. In order to achieve the best photo crowdsensing performance (deliver ratio or average delay), we should make a decision about which photo to be with a higher priority. (2) If a new photo comes to a user’s storage full of photos, in order to achieve the best photo crowdsensing performance, we must make a decision about which photo to delete between the persistent photos and the new incoming photo.

Here, we present a storage management strategy in mobile phones for photo crowdsensing, which first expresses the photo crowdsensing performances (delivery ratio and average delay) as a function of variables. The utility for each photo is achieved through the derivative value of the photo crowdsensing performance. If the transmission speed for the connections is not enough to send all the photos stored in the storage, the user will copy photos from high to low for the utility of each photo. If storage is overflowed, the user will make a decision about which photo to delete according to the utility, in order to achieve the best photo crowdsensing performance.

We first define several notations. For example, we regard the delivery ratio as the photo crowdsensing performance (average delay has a similar derivation). $P_{i}$ is the probability to finish delivering photo *i*, $n_{i}$ is the number of photo *i* in users’ storages when the time is $t_{i}$. $U_{i}$ is the utility of photo *i* measured by the impact of sending or deleting the photo on the performance. Then, the following situations are considered:

$\left\{\begin{array}{cc} \Delta (n_{i})=1 & \mathrm{copy}\;\mathrm{photo}\;i.\\ \Delta (n_{i})=0 & \mathrm{do}\;\mathrm{nothing}.\\ \Delta (n_{i})=-1 & \mathrm{delete}\;\mathrm{existing}\;\mathrm{photo}\;i. \end{array}\right.$

Therefore, $\Delta P_{i}=U_{i}\Delta \left(n_{i}\right)$, $U_{i}=\frac{\partial P_{i}}{\partial n_{i}}$.

According to the above descriptions, we schedule the photos in the photo’s decreasing order, and photos in high order are copied when there is communication opportunity. If the storage is overflowed, then we will delete the photos with the lowest utility.

## 4. Proposed Storage Management Framework

**Figure 3:**
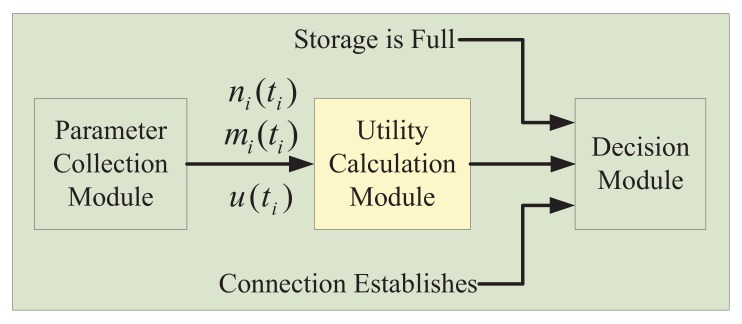
The storage management framework.

Figure 3 uses a readily comprehensible view to describe the storage management framework of mobile photo crowdsensing, including three modules and transitions among them. The parameter collection module is used to collect the following three parameters $n_{i}\left(t_{i}\right)$, $m_{i}\left(t_{i}\right)$, and $u(t_{i})$, which could be regarded as the inputs of the utility calculation module. The utility calculation module is the main part of the framework. The decision of sending or deleting the photos in storage is achieved according to the storage occupancy status and the utility value of the photos. Then, the three modules are described in detail as follows.

### 4.1. Parameter Collection Module

In order to calculate the per-photo utility, which is used to decide the sending and deleting priority, the network parameters are exchanged among users, which include the following data: (1) $n_{i}\left(t_{i}\right)$, which is the number of photo *i* when the time is $t_{i}$. (2) $m_{i}\left(t_{i}\right)$, which is the number of devices for every carry photo *i* when the time is $t_{i}$. (3) $u(t_{i})$, which is the number of users that have been in any PoI before the photo *i*’s elapsed time $t_{i}$. The exchange process ensures that each user could estimate the above three parameters through collecting and exchanging the information of the other users. Section 4 describes in detail how the parameters are collected.

### 4.2. Utility Calculation Module

In this subsection, we attempt to calculate the photo’s utility; in other words, the following problem is addressed: $m_{i}\left(t_{i}\right)$, $n_{i}\left(t_{i}\right)$, and $u(t_{i})$, and limited storage space for supporting epidemic photo exchanging strategy are known, we should find a suitable strategy about whether to receive the new photo or just reject the coming photo, in order to optimize our performance goals. In Section 4, we introduce in detail the process to calculate the photo’s utility. Two optimization goals are regarded as the purposes in terms of photo crowdsensing.

### 4.3. Sending and Deleting Decisions

**Figure 4:**
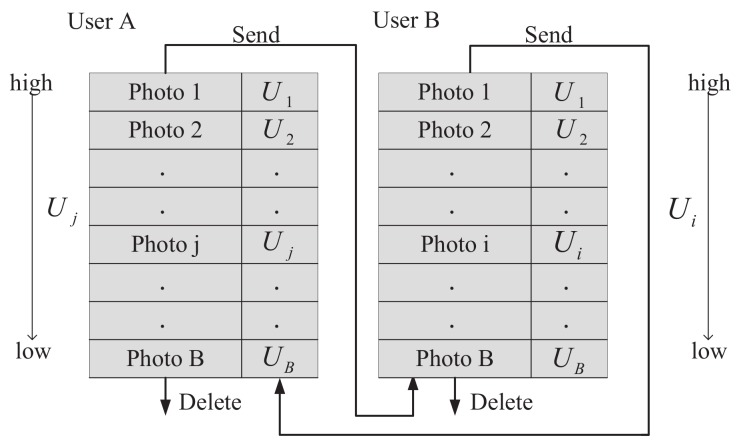
The management process in the user’s storage.

With the per-photo utility, the user first sorts the photos of its storage from high to low. When the storage is overflowed, the photo of lower utility could be first deleted from the storage; when there is a communication probability, the photos of higher utility could be copied first to an encounter. Figure 4 describes the sending and deleting processes: if the photo *j*’s utility $U_{j}$ in user *A*’s storage is the highest. In addition, it is higher than $U_{B}$ of photo *i* at user *B*, which is the lowest in user *B*. Then, photo *i* is deleted and a copy of photo *j* enters user *B*’s storage when users *A* and *B* encounter each other. The detailed decision algorithm is shown in Algorithm 1, called Storage Management for Photo Crowdsensing (SMPC).

**Algorithm 1** SMPC
**Input:**

  Capacity of photos in a user’s storage: *n*  ID for a new arriving photo: *m*

**Output:**

  Sending photo: $ID_{S}$, Deleting photo: $ID_{D}$
1:**for***i* =1 to *n* **do**2: Calculate $U_{i}$3:Sort $U_{i}$ in a progressive increase manner  4:Search for the highest $U_{h}$, and assign *h* to $ID_{S}$  5:Search for the lowest $U_{l}$, and assign *l* to $ID_{D}$  6:**if** there is a connection **then**7: **return** $ID_{S}$
8:**if** storage is overflowed **then**9: Calculate $U_{m}$  10: **if** $U_{m}<U_{l}$ **then**
11:  assign *m* to $ID_{D}$12: **return** $ID_{D}$



## 5. Utility-Based Storage Management Strategy

### 5.1. Mobility Pattern in Mobile Crowdsensing

In the photo crowdsensing environment, users mainly utilize occasional communication opportunities to copy photos. Therefore, the intermeeting time among users and that between users and PoIs will make an important impact on delivery ratio and average delay of photos. Focusing on this, we first give the following two definitions:

**Definition** **1.**  *Intermeeting time $E_{1}$ focuses on a pair of users; it defines the time span between the last connection and the current connection.*

**Definition** **2.**  *Intermeeting time $E_{2}$ focuses on a PoI and a user; it also defines the time span between the last connection and the current connection.*

Recent research [42] proves that the intermeeting time satisfies an exponential distribution in some mobility patterns, such as random walk and random-waypoint because the simulations of this paper are tested in the random-waypoint scenario and three real-world traces (*roma/taxi trace set*, *epfl trace set*, and *geolife trace set*). The first group of simulations are about the distribution of the intermeeting times for the four scenarios above, in order to see if they can match the exponential distribution.

**Figure 5:**
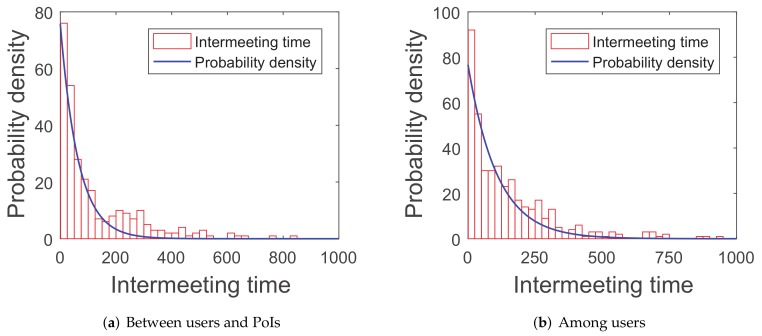
Random-waypoint mobility pattern.

**Figure 6:**
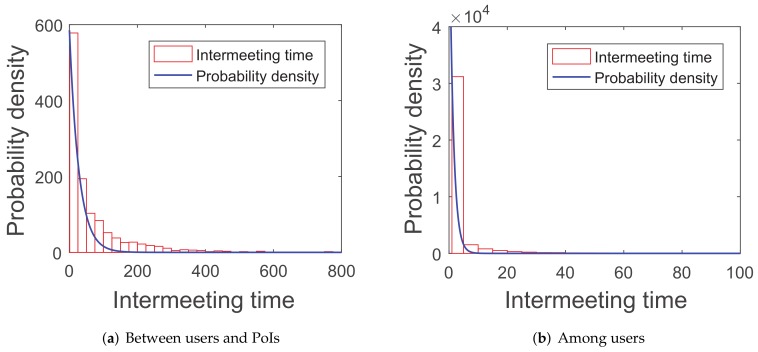
Roma/taxi trace set.

**Figure 7:**
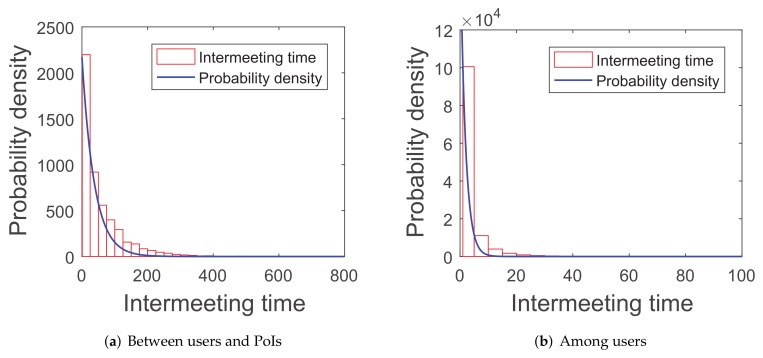
Epfl trace set.

**Figure 8:**
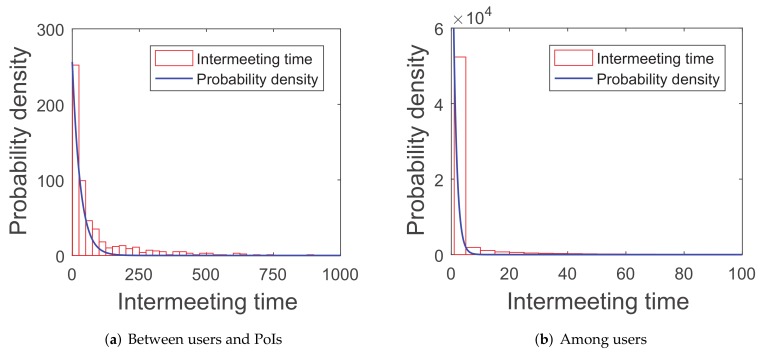
Geolife trace set.

As shown in Figure 5, Figure 6, Figure 7 and Figure 8, the intermeeting times approximately match the exponential distribution for the above situations: $f\left(x\right)=\lambda e^{-\lambda x}\;\left(x\geq 0\right)$. $\lambda_{1}$ and $\lambda_{2}$ are shown in Table 1; then, we have $\lambda_{1}=\frac{1}{E_{1}}$ and $\lambda_{2}=\frac{1}{E_{2}}$.

### 5.2. Maximization of Delivery Ratio

The probability to finish delivering photo *i*: $P_{i}$ could be calculated through $P_{t_{i}}$, which is defined as probability to finish delivering photo *i* to PoIs when the time is $t_{i}$, and $P_{r_{i}}$, which is defined as probability to finish delivering the undelivered photo *i* when the remaining time is $r_{i}$. Then, $P_{i}$ could be calculated as follows:

(1) $$\begin{array}{cc} P_{i} & =\left(1-P_{t_{i}}\right)P_{r_{i}}+P_{t_{i}} \end{array}$$

The elapsed time for photo *i* since its born date is $t_{i}$. At time $t_{i}$, we assume that $m_{i}\left(t_{i}\right)$ users (excluding source) have carried a copy of photo *i*. Moreover, $u(t_{i})$ represents the users that have ever been in any PoI before the current time $t_{i}$. The delivery ratio could be regarded as the probability that at least one user is not only among $m_{i}\left(t_{i}\right)$ users but also in $u(t_{i})$ users. Therefore, the probability $P(t_{i})$ can be shown as Equation (2):

(2) $$\begin{array}{cc} \; & P_{t_{i}}=1-\frac{C_{N}^{u(t_{i})}C_{N-u(t_{i})}^{m_{i}\left(t_{i}\right)}}{C_{N}^{u(t_{i})}C_{N}^{m_{i}\left(t_{i}\right)}}=1-\frac{C_{N-u(t_{i})}^{m_{i}\left(t_{i}\right)}}{C_{N}^{m_{i}\left(t_{i}\right)}}=1-\frac{\left(N\begin{array}{c} - \end{array}u\left(t_{i}\right)\right)\left(N\begin{array}{c} - \end{array}u\left(t_{i}\right)\begin{array}{c} - \end{array}1\right)\cdots (N\begin{array}{c} - \end{array}u\left(t_{i}\right)\begin{array}{c} - \end{array}\left(m_{i}\left(t_{i}\right)\begin{array}{c} - \end{array}1\right))}{N\left(N-1\right)\cdots (N\begin{array}{c} - \end{array}\left(m_{i}\left(t_{i}\right)\begin{array}{c} - \end{array}1\right))} \end{array}$$

Equation (2) is reasonable because the following two characters are achieved: (1) when $m_{i}\left(t_{i}\right)$ increases, $P(t_{i})$ also increases, which is shown in Equation (3). This matches our common sense: more users that have seen photo *i* will lead to a higher delivery ratio at time $t_{i}$. (2) when $u(t_{i})$ increases, $P(t_{i})$ also increases, which is shown in Equation (4). This also makes sense because more users that have ever been in any PoI before time $t_{i}$ will also lead a higher delivery ratio. Therefore, Equation (2) could precisely reflect the delivery probability of photo *i*:

(3) $$\begin{array}{c} \frac{C_{N-u(t_{i})}^{m_{i}\left(t_{i}\right)}}{C_{N}^{m_{i}\left(t_{i}\right)}}>\frac{C_{N-u(t_{i})}^{m_{i}\left(t_{i}\right)+1}}{C_{N}^{m_{i}\left(t_{i}\right)+1}} \end{array}$$

(4) $$\begin{array}{c} \frac{C_{N-u(t_{i})}^{m_{i}\left(t_{i}\right)}}{C_{N}^{m_{i}\left(t_{i}\right)}}>\frac{C_{N-u(t_{i})-1}^{m_{i}\left(t_{i}\right)}}{C_{N}^{m_{i}\left(t_{i}\right)}} \end{array}$$

$P_{r_{i}}$ as shown in Equation (1) has been described in Table 1. The calculation of $P_{r_{i}}$ is the major difficulty of the utility calculation model. First of all, we pay attention to the relationship between $n_{i}\left(t\right)$ and the time *t*. Taking the ordinary-differential-equation model [29] into consideration, we achieve the following:

(5) $$\begin{array}{cc} \frac{dn_{i}\left(t\right)}{dt} & =\lambda_{1}n_{i}\left(t\right)\left[N-n_{i}\left(t\right)\right], \end{array}$$

where the parameter $\lambda_{1}=\frac{1}{E_{1}}$. Through solving Equation (5), we achieve Equation (6) as follows:

(6) $$\begin{array}{c} n_{i}\left(t\right)=\frac{Nn_{i}\left(0\right)}{n_{i}\left(0\right)+\left[N-n_{i}\left(0\right)\right]e^{-\lambda_{1}Nt}}. \end{array}$$

Then, when the time is $t_{i}+r_{i}$, the number of users could be calculated as follows:

(7) $$\begin{array}{c} n_{i}\left(t_{i}+r_{i}\right)=\frac{Nn_{i}\left(t_{i}\right)}{n_{i}\left(t_{i}\right)+\left[N-n_{i}\left(t_{i}\right)\right]e^{-\lambda_{1}Nr_{i}}} \end{array}$$

Next, we attempt to calculate $P_{r_{i}}$. The equation $1-P_{r_{i}}$ is the situation that photo *i* does not finish delivering when the time is $t_{i}$, and can not finish delivering in the rest of TTL: $r_{i}$$\left(r_{i}=TTL-t_{i}\right)$. In other words, $1-P_{r_{i}}$ is the situation in which not only can the $n_{i}\left(r_{i}\right)$ users with photo *i* not be the destination PoI during $r_{i}$, but also the new users with photo *i* can not deliver the photo to any PoI before $r_{i}$. Then, the following equation is achieved:

(8) $$\begin{array}{cc} 1-P_{r_{i}} & =e^{-\lambda_{2}n_{i}\left(t_{i}\right)r_{i}}\prod_{t=0}^{r_{i}}e^{-\lambda_{2}n_{i}^{\prime }\left(t_{i}+t\right)\left(r_{i}-t\right)}=\frac{e^{-\lambda_{2}n_{i}\left(t_{i}\right)r_{i}}\prod_{t=0}^{r_{i}}e^{-\lambda_{2}n_{i}^{\prime }\left(t_{i}+t\right)\left(r_{i}\right)}}{\prod_{t=0}^{r_{i}}e^{-\lambda_{2}n_{i}^{\prime }\left(t_{i}+t\right)\left(t\right)}}=\frac{e^{-\lambda_{2}n_{i}\left(t_{i}+r_{i}\right)r_{i}}}{e^{-\lambda_{2}\int_{0}^{r_{i}}n_{i}^{\prime }\left(t_{i}+t\right)tdt}} \end{array}$$

The quantity $n_{i}\left(t_{i}+r_{i}\right)$ in Equation (8) can be achieved as follows:

(9) $$\begin{array}{cc} \int_{0}^{r_{i}}n_{i}^{\prime }\left(t_{i}+t\right)tdt & =\int_{0}^{r_{i}}tdn_{i}\left(t_{i}+t\right)={|}_{0}^{r_{i}}tn_{i}\left(t_{i}+t\right)-\int_{0}^{r_{i}}n_{i}\left(t_{i}+t\right)dt\\ \; & =r_{i}n_{i}\left(t_{i}+r_{i}\right)-\left(Nr_{i}+\frac{\ln[n_{i}\left(t_{i}\right)-n_{i}\left(t_{i}\right)e^{-\lambda_{1}Nr_{i}}+Ne^{-\lambda_{1}Nr_{i}}]}{\lambda_{1}}\right)+\frac{\ln(N)}{\lambda_{1}} \end{array}$$

By combining Equations (7)–(9), the solution of $P_{r_{i}}$ is easy to achieve:

(10) $$\begin{array}{cc} 1-P_{r_{i}} & =\frac{N^{\frac{\lambda_{2}}{\lambda_{1}}}}{e^{-\lambda_{2}Nr_{i}}{\left[n_{i}\left(t_{i}\right)-n_{i}\left(t_{i}\right)e^{-\lambda_{1}Nr_{i}}+Ne^{-\lambda_{1}Nr_{i}}\right]}^{\frac{\lambda_{2}}{\lambda_{1}}}} \end{array}$$

Then, the final equation $P_{i}$ is shown as follows:

(11) $$\begin{array}{cc} \; & P_{i}=1\begin{array}{c} - \end{array}\frac{C_{N\begin{array}{c} - \end{array}u\left(t_{i}\right)}^{m_{i}\left(t_{i}\right)}}{C_{N}^{m_{i}\left(t_{i}\right)}}+\frac{C_{N\begin{array}{c} - \end{array}u\left(t_{i}\right)}^{m_{i}\left(t_{i}\right)}}{C_{N}^{m_{i}\left(t_{i}\right)}}\left(1\begin{array}{c} - \end{array}\frac{N^{\frac{\lambda_{2}}{\lambda_{1}}}}{e^{\begin{array}{c} - \end{array}\lambda_{2}Nr_{i}}{\left[n_{i}\left(t_{i}\right)\begin{array}{c} - \end{array}n_{i}\left(t_{i}\right)e^{\begin{array}{c} - \end{array}\lambda_{1}Nr_{i}}\begin{array}{c} + \end{array}Ne^{\begin{array}{c} - \end{array}\lambda_{1}Nr_{i}}\right]}^{\frac{\lambda_{2}}{\lambda_{1}}}}\right) \end{array}$$

Then, the utility for photo *i* can be derived through the following equation:

(12) $$\begin{array}{cc} \; & \Delta \left(P_{i}\right)=\frac{d\left(1-P_{t_{i}}\right)P_{r_{i}}}{dn_{i}\left(t_{i}\right)}=\frac{C_{N\begin{array}{c} - \end{array}u\left(t_{i}\right)}^{m_{i}\left(t_{i}\right)}}{C_{N}^{m_{i}\left(t_{i}\right)}}\frac{\lambda_{2}}{\lambda_{1}}\left(1-e^{-\lambda_{1}Nr_{i}}\right)\frac{N^{\frac{\lambda_{2}}{\lambda_{1}}}}{e^{\begin{array}{c} - \end{array}\lambda_{2}Nr_{i}}{\left[n_{i}\left(t_{i}\right)\begin{array}{c} - \end{array}n_{i}\left(t_{i}\right)e^{\begin{array}{c} - \end{array}\lambda_{1}Nr_{i}}\begin{array}{c} + \end{array}Ne^{\begin{array}{c} - \end{array}\lambda_{1}Nr_{i}}\right]}^{\frac{\lambda_{2}}{\lambda_{1}}+1}}\Delta n_{i}\left(t_{i}\right) \end{array}$$

The storage management proposed here is to optimize the delivery ratio performance in the photo crowdsensing. If a photo *i* is copied successfully, the photo *i*’s copies add one [$\Delta n_{i}\left(t_{i}\right)=+1$]; if nothing is done for photo *i*, the number of photo *i*’s copies does not change [$\Delta n_{i}\left(t_{i}\right)=0$]; if photo *i* is deleted from a user’s storage, the number of photo *i* subtracts one [$\Delta n_{i}\left(t_{i}\right)=-1$]. Therefore, the utility of photo *i* is just $\Delta (P_{i})$. The delivery ratio per-photo utility is achieved:

(13) $$\begin{array}{cc} \; & U_{i}=\frac{C_{N\begin{array}{c} - \end{array}u\left(t_{i}\right)}^{m_{i}\left(t_{i}\right)}}{C_{N}^{m_{i}\left(t_{i}\right)}}\frac{\lambda_{2}}{\lambda_{1}}\left(1-e^{-\lambda_{1}Nr_{i}}\right)\frac{N^{\frac{\lambda_{2}}{\lambda_{1}}}}{e^{\begin{array}{c} - \end{array}\lambda_{2}Nr_{i}}{\left[n_{i}\left(t_{i}\right)\begin{array}{c} - \end{array}n_{i}\left(t_{i}\right)e^{\begin{array}{c} - \end{array}\lambda_{1}Nr_{i}}\begin{array}{c} + \end{array}Ne^{\begin{array}{c} - \end{array}\lambda_{1}Nr_{i}}\right]}^{\frac{\lambda_{2}}{\lambda_{1}}+1}}. \end{array}$$

### 5.3. Minimization of Average Delay

In this section, focusing on the performance of average delay for time-sensitive photo crowdsensing [43], we assume that the deadlines for all the photos are so long that a close-to-1 delivery ratio could be achieved. The following derivation gives the way to optimize the performance of the average delay for all the photos.

Delivery delay for photo *i* is defined as $D_{i}$. If photo *i* has been successfully transmitted to a PoI at time $t_{i}$, the expected delivery delay for photo *i* is defined as $D_{t_{i}}$. Otherwise, the expected delivery delay for photo *i* within the TTL is $D_{r_{i}}$:

(14) $$\begin{array}{c} D_{i}=P_{t_{i}}D_{t_{i}}+\left(1-P_{t_{i}}\right)D_{r_{i}} \end{array}$$

It is not difficult to find that, $D_{t_{i}}$ could be regarded as 0 if the photo *i* has already been delivered. The earliest time for the first photo *i* to be delivered to PoI is an exponential distribution with average value: $\frac{1}{n_{i}\left(t_{i}\right)\lambda_{2}}$. Thus, $D_{r_{i}}=t_{i}+\frac{1}{n_{i}\left(t_{i}\right)\lambda_{2}}$. Then, we could achieve the expected average delay ($D_{i}$),

(15) $$\begin{array}{c} D_{i}=\left(1-\frac{C_{N\begin{array}{c} - \end{array}u\left(t_{i}\right)}^{m_{i}\left(t_{i}\right)}}{C_{N}^{m_{i}\left(t_{i}\right)}}\right)*0+\frac{C_{N\begin{array}{c} - \end{array}u\left(t_{i}\right)}^{m_{i}\left(t_{i}\right)}}{C_{N}^{m_{i}\left(t_{i}\right)}}\left(t_{i}+\frac{1}{n_{i}\left(t_{i}\right)\lambda_{2}}\right) \end{array}$$

Now, we differentiate $D_{i}$ in terms of $n_{i}\left(t_{i}\right)$ to propose a strategy that maximizes the per-size increase in $D_{i}$,

(16) $$\begin{array}{cc} \; & \Delta \left(D_{i}\right)=-\frac{C_{N\begin{array}{c} - \end{array}u\left(t_{i}\right)}^{m_{i}\left(t_{i}\right)}}{C_{N}^{m_{i}\left(t_{i}\right)}}\frac{1}{n_{i}{\left(t_{i}\right)}^{2}\lambda_{2}}\Delta n_{i}\left(t_{i}\right) \end{array}$$

The best deleting or sending decision will be the one that maximizes $\left|\Delta (D_{i})\right|$. We obtain the following equation for calculating per-photo average delay utility:

(17) $$\begin{array}{cc} \; & U_{i}=\frac{C_{N\begin{array}{c} - \end{array}u\left(t_{i}\right)}^{m_{i}\left(t_{i}\right)}}{C_{N}^{m_{i}\left(t_{i}\right)}}\frac{1}{n_{i}{\left(t_{i}\right)}^{2}\lambda_{2}} \end{array}$$

Because the utility for average delay is different with the one for delivery ratio, the two performances could not be optimized concurrently.

### 5.4. Parameters Collection

**Figure 9:**
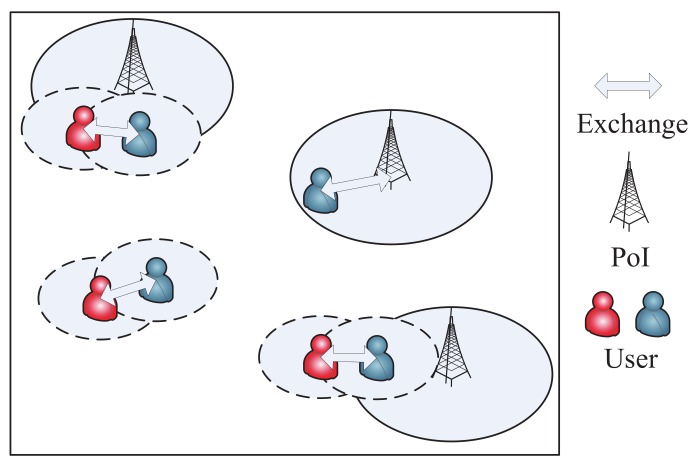
The communication conditions among the users and PoIs. The PoIs and the users are regarded as the same; they collect parameters and exchange with each other.

As described in Section 3-A, three parameters $n_{i}\left(t_{i}\right)$, $m_{i}\left(t_{i}\right)$, and $u(t_{i})$ need to be collected in order to calculate the per-photo utility. Some specialists [43] consider that all the uncertain variables could be achieved by control center. However, actually the design is impractical in the mobile photo crowdsensing. Therefore, we propose a parameter collection way through regarding the PoIs as the common users. Then, all the users collect and exchange their own storage history and mobility history. The communication conditions among the users and PoIs are shown in Figure 9.

#### 5.4.1. Collection of $m_{i}\left(t_{i}\right)$

**Figure 10:**
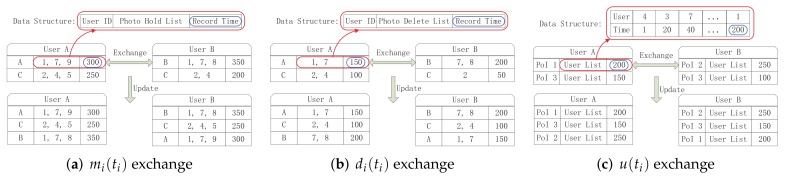
Parameter collection process to estimate $m_{i}\left(t_{i}\right)$, $d_{i}\left(t_{i}\right)$, and $u(t_{i})$.

Every user holds a table as shown in Figure 10a. Obviously, the space occupied by a table is far smaller than the photo size. The $m_{i}\left(t_{i}\right)$ table includes all photos ever stored, and updating time, which is updated when two tables exchange records; the update process is shown in Figure 10a. It is worth noting that nobody except the initial user could alter the record time; only when a new photo enters its storage could the record then be modified. When two users with the same records encounter each other, they will not exchange photos; however, they could update the newest record time with each. In this way, all the users can estimate $m_{i}\left(t_{i}\right)$ for photo *i*.

#### 5.4.2. Collection of $n_{i}\left(t_{i}\right)$

According to the definition of $d_{i}\left(t_{i}\right)$ in Table 1, $m_{i}\left(t_{i}\right)$ and $n_{i}\left(t_{i}\right)$ can be associated through Equation (18). Therefore, the problem turns to collect $d_{i}\left(t_{i}\right)$, which is easier for us to collect compared with $n_{i}\left(t_{i}\right)$. The collection process of $d_{i}\left(t_{i}\right)$ is shown in Figure 10b, which is similar to that of $m_{i}\left(t_{i}\right)$. Every node maintains a data structure including user id, deleted photo list, and record time. The deleted list contains all deleted photos; when users encounter each other, they exchange and update the records. It is worth noting that users will not receive the same photo twice [44]:

(18) $$\begin{array}{cc} n_{i}\left(t_{i}\right) & =m_{i}\left(t_{i}\right)+1-d_{i}\left(t_{i}\right) \end{array}$$

#### 5.4.3. Collection of $u(t_{i})$

Every PoI records the ever-been user list, which includes the user id and the entering time. The data structure is shown in Figure 10c. When a user enters a PoI, the PoI adds the user to the ever-been user list. At the same time, the user takes the records of the PoI and exchanges them with the other users. The exchange process is also shown in Figure 10c. $u(t_{i})$ is the number of users that have ever been in any PoI in photo *i*’s elapsed time $t_{i}$. After a period of time, every user has the ever-been user list for all the PoIs, so they could calculate the number of users that have ever been in the PoI before the elapsed time $t_{i}$. Therefore, $u(t_{i})$ is achieved.

Through the above parameter collection methods, $n_{i}\left(t_{i}\right)$, $m_{i}\left(t_{i}\right)$, and $u(t_{i})$ could be calculated through every user’s collected records. Then, every user could achieve the utilities of all the photos stored in its storage. Finally, they could make sending and deleting decisions according to the utility. Thus, a storage management strategy in mobile phones for photo crowdsensing is proposed.

## 6. Performance Evaluation

### 6.1. The Traces Used and Settings

The random-waypoint mobility pattern and three real traces, *roma/taxi trace set* [45], *epfl trace set* [46], and *geolife trace set* [47,48], are used to prove the performances of the proposed strategies. Random-waypoint mobility pattern requires each user to keep doing the same actions in a fixed area, walking directly to a certain destination, which is randomly ensured. The *roma/taxi* records 320 users, who work in Rome, Italy. GPS could help record their positions, and the central server could collect all the users’ positions. The *epfl* is collected in San Francisco, CA, USA. It is the GPS data from about 500 taxis collected over 30 days in the San Francisco Bay Area. The *geolife* has 17,621 traces, which move about 1.2 million kilometers and continue for about 48,000+ hours.

**Figure 11:**
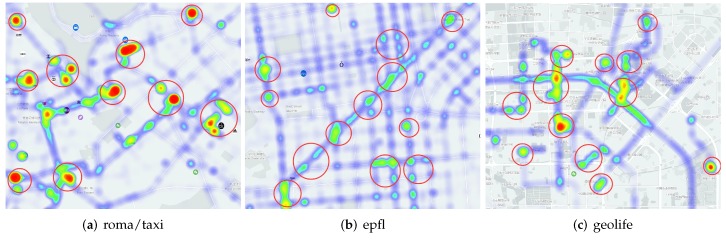
The PoI areas in Baidu map of the three real-world data sets.

We filter the above three real-world traces to remove some anomaly trajectories (intermittent or faraway traces). Baidu map is used to carry the GPS traces. Through JavaScript API, we draw the map and corresponding thermodynamic chart (Figure 11), while the PoIs of random-waypoint are set randomly. The simulation parameters in this network environment are shown in Table 2.

### 6.2. Strategies and Performances in Comparison

To test the proposed storage management strategies, our simulations are divided into the following two parts: (1) performances of the proposed storage management strategies and (2) distribution of utility.

For the first part, we mainly consider the following two proposed management strategies: Storage Management strategies in mobile phones for Photo Crowdsensing to maximize delivery Ratio (SMPC-R) and Storage Management strategies in mobile phones for Photo Crowdsensing to minimize average Delay (SMPC-D). SMPC-R refers to the delivery ratio utility as the priority to send and delete photos, while SMPC-D uses the average delay utility. We attempt to prove that SMPC-R achieves the highest delivery ratio and SMPC-D gets the lowest average delay compared with the other four storage management strategies [29]: (1) DL-Delete Last (or ”Delete tail”) deletes the just comer photo, (2) DF-Delete Front deletes the photo staying in the buffer for the longest time when the buffer is full, (3) DO-Delete Oldest deletes the photo that has lived for the longest time, and (4) DY-Delete Youngest deletes the photo that is the youngest.

For the second part, we turn our attention to the utility distributions. We want to test whether the distributions calculated in this paper for SMPC-R and SMPC-D are sensitive to different buffer overflown levels. Moreover, we also pay attention to how SMPC performs in the different congestion regimes and whether the resulting utility shape could be approximated by simpler policies.

The following two performances are mainly taken into consideration:

1. Delivery ratio: successfully delivered photo number divided by total number of photos.
2. Average delay: required average time for the photos that have been delivered.

### 6.3. Simulation Results

#### 6.3.1. Performances of SMPC-R and SMPC-D

**Figure 12:**
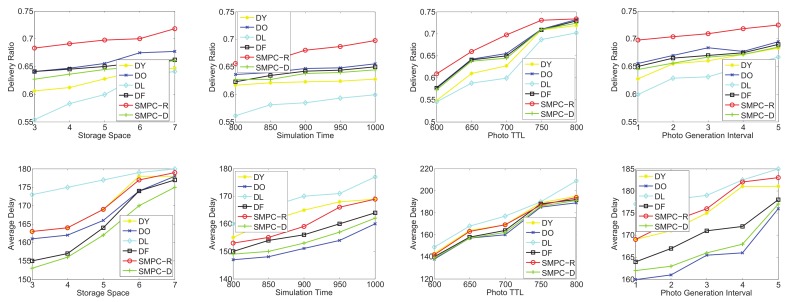
Performance comparisons on the random-waypoint mobility pattern: delivery ratio and average delay.

**Figure 13:**
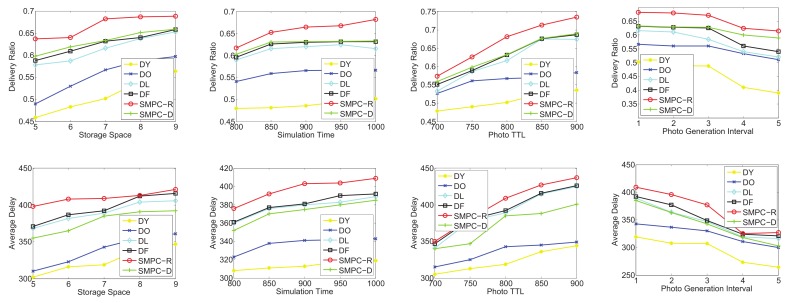
Performance comparisons on the *roma/taxi trace set*: delivery ratio and average delay.

**Figure 14:**
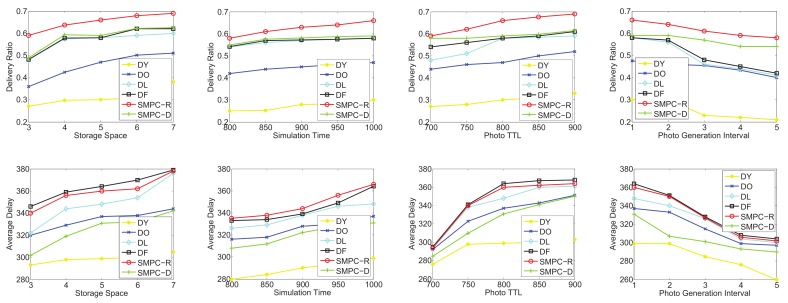
Performance comparisons on the *epfl trace set*: delivery ratio and average delay.

**Figure 15:**
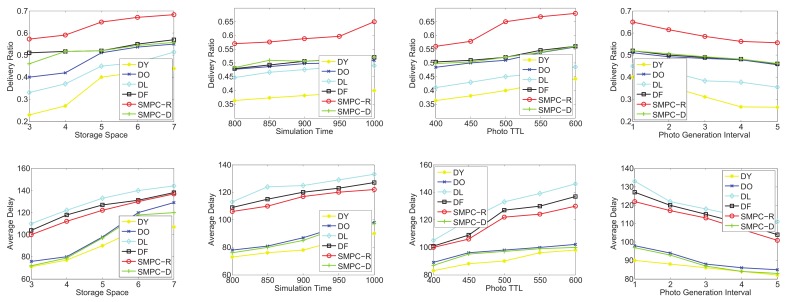
Performance comparisons on the *geolife trace set*: delivery ratio and average delay.

To test the utilities of SMPC-R and SMPC-D, we focus on four sets of simulations in terms of the random-waypoint mobility pattern and the three traces: *roma/taxi*, *epfl*, and *geolife*, respectively. SMPC-R refers to the utility of Equation (13) as the priority to decide which photo to send or delete, while SMPC-D considers the utility of Equation (17) as the priority. We consider that the following four factors could influence the network performances: storage space, simulation time, photo TTL, and photo generation interval. Therefore, the performances as a function of the storage space, simulation time, photo TTL, and generation interval are shown in Figure 12, Figure 13, Figure 14 and Figure 15 for the four data sets, respectively.

As seen in Figure 12, the delivery and delay performances in terms of storage space, simulation time, photo TTL, and generation interval are tested. The ranking of the delivery ratio performances is SMPC-R>DO>DF>SMPC-D>DY>DL, which is reasonable because SMPC-R refers to the utility, which is used to maximize the delivery ratio, as the priority to send or delete photos. In other words, SMPC-R decides whether to send or delete a photo, aiming to maximize the delivery ratio of all the photos. Thus, SMPC-R gets the best delivery performance. Moreover, DO and DF achieve a higher delivery ratio than that of SMPC-D, DY, and DL, which makes sense because DO and DF delete the photos in old age, and the new photos have more opportunities to be stored in storage and delivered to the PoIs.

It is worth noting that delivery ratios grow up with the increase of the storage space for all the storage management strategies. This makes sense and is easy to understand. Moreover, with the growth of simulation time and photo TTL, the delivery ratio also appears as an increasing trend because a longer simulation time or photo TTL leads to a higher probability for delivering the photos. In addition, a higher photo generation interval also means a lower congestion situation, which is similar to that of a larger storage space. Therefore, the delivery ratio also increases with the growth of the photo generation interval for all the storage management strategies.

Note that we also test the average delay as a function of storage space, simulation time, photo TTL, and generation interval (as shown in Figure 12). The ranking of delay performances is DL>DY>SMPC-R>DO>DF>SMPC-D, which is reasonable because SMPC-D considers the utility, which is used to minimize the average delay, as the priority to send or delete photos. In other words, SMPC-D decides to send the photo that has the highest influence on decreasing the average delay, and decides to delete the the photo that has the lowest influence on increasing the average delay. Thus, the average delay of SMPC-D is the lowest. Moreover, DL and DY achieve the highest average delay out of all the other storage management strategies because DY and DL delete the new photos, so the average elapsed time of the successfully delivered photos is the highest.

Most importantly, the average delay increases with the growth of the storage space for all the storage management strategies. This is because more storage space leads to a larger space for the photo to be stored, and some photos deleted due to storage overflowing could be delivered when the storage becomes larger. Thus, the average delay of all the successfully delivered photos becomes larger. Moreover, as the simulation time and photo TTL increase, the average delay also appears as an increasing trend because a longer simulation time or photo TTL lead to a higher elapsed time to deliver the photos. In addition, a higher photo generation interval also means a larger storage space, which is explained in the previous paragraph. Therefore, the average delay also increases with the growth of the photo generation interval.

As shown in Figure 13, in the second group simulation, we test the performance comparisons (delivery ratio and average delay) on the on the *roma/taxi trace set* as a function of storage space, simulation time, photo TTL, and generation interval. Obviously, SMPC-R also achieves the highest delivery ratio in terms of storage space, simulation time, photo TTL, and generation interval, while DY also gets the lowest delivery ratio. Actually, DY just deletes the newest photo (the youngest photo) in each mobile phone’s storage. Therefore, the delivery ratio of DY is not good in photo crowdsensing. Intuitively, we should protect the new photos (just taken) from being deleted, and delete the old photos when the storage is overflowed. In this way, the photo crowdsensing performance could be better.

Similar results to that of Figure 12 are shown in Figure 13; the average delay of all the storage management strategies also appears in an upward trend along with the increase of the storage space, which is easy to understand. However, SMPC-D achieves a higher average delay compared with DY and DO. Through analysis, in the delivery ratio simulations of *roma/taxi trace set*, DY and DO achieve the lowest results; the definition of average delay is required average time for the photos that have been finished delivering. Thus, the photos that have been delivered have a shorter delivery time, and there are a larger number of undelivered photos. The above reasons explain why SMPC-D does not achieve the lowest average delay. We omit detailed descriptions of Figure 14 and Figure 15 because their simulation results are similar to those of Figure 12 and Figure 13.

In conclusion, SMPC-R achieves the highest delivery ratio and SMPC-D achieves the lowest average delay.

#### 6.3.2. Distribution of Utility

In this subsection, we focus on the research in terms of the utility distributions. We attempt to show the changes of SMPC-R and SMPC-D utilities along with time *t* in the different congestion regimes. We also consider whether the resulting utility shape is related to the different congestion levels.

**Figure 16:**
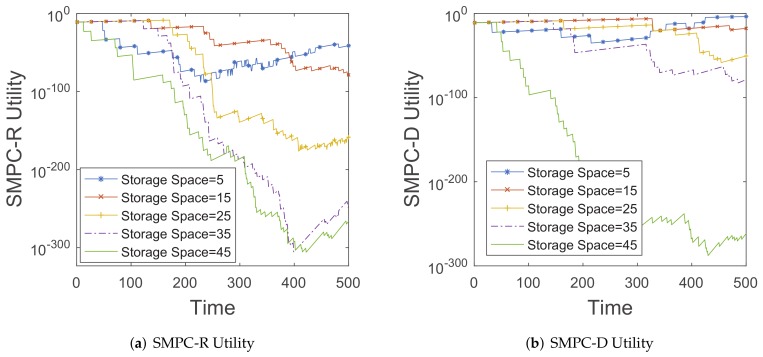
The changes of SMPC-R and SMPC-D utilities along with time *t* in the different storage spaces.

We focus again on the *roma/taxi trace set*. First, we change the storage space from 5 to 45, corresponding to the different congestion regimes. In Figure 16, we plot the utility for maximizing the delivery ratio (SMPC-R) and utility for minimizing the average delay (SMPC-D) described in Sections IV-B and IV-C, as a function of time *t*. Obviously, the utility distribution has a non-trivial shape, resulting in a complex photo sending and deleting strategy in the mobile phone’s storage space.

As shown in Figure 16a, when the storage space is 5, the scenario of a mobile phone is considered as a high congestion. We could see that the SMPC-R utility appears as a complex change along with time *t*. This precisely helps explain why simple delete and send strategies (e.g., Delete Youngest, Delete Oldest, Delete Front, or Delete Finally, etc.) lead to incorrect decisions during congestion and perform worse than the SMPC-R. When the storage space is 45, the scenario of the mobile phone is considered as a low congestion. We could see that the shape of SMPC-R utility appears as a downward trend along with time *t*. This suggests that the storage management strategy in low congestion regimes could be approximated by the simpler ”Delete Oldest photo” policy, which does not require collecting and exchanging parameters among users.

## 7. Conclusions

We have looked into the problem of storage management in mobile phones for photo crowdsensing. First, we consider the users with stored photos as the sources, and also consider the PoIs as the destinations. The epidemic exchanging process is implemented to upload the photos from the sources to the destinations. Then, taking the limited storage of mobile phones into consideration, we propose two utility-based storage management strategies in mobile phones for photo crowdsensing: one is for maximizing delivery ratio (SMPC-R) and the other one is for minimizing average delay (SMPC-D). The storage management strategy makes the sending/deleting decisions according to the photo’s utility, which is calculated by measuring the impact of copying or deleting the photo on the considered performances. We have done simulations based on the random-waypoint model and three real traces: *roma/taxi*, *epfl*, and *geolife*. The results show that, compared with other storage management strategies, SMPC-R gets the highest delivery ratio and SMPC-D achieves the lowest average delay. 

## Figures and Tables

**Table 1:** Main Symbols of this paper

Symbol	Meaning
*N*	the number of users minus one
$TTL_{i}$	uploaded deadline for the sensing photo *i*
$r_{i}$	remaining live time for photo *i*
$t_{i}$	elapsed time for photo *i* from its generation time
	to the current time ($t_{i}=TTL_{i}-r_{i}$)
$n_{i}\left(t_{i}\right)$	copy number of photo *i* in the storages when the time is $t_{i}$
$d_{i}\left(t_{i}\right)$	copy number of deleted photo *i* when the time is $t_{i}$
$m_{i}\left(t_{i}\right)$	number of devices ever carry photo *i* when the time is $t_{i}$
$u(t_{i})$	number of users that have ever been in any PoI
	in photo *i*’s elapsed time $t_{i}$
$\lambda_{1}$	variable for intermeeting time distribution among users
$E_{1}$	expected intermeeting time between users ($E_{1}=\frac{1}{\lambda_{1}}$)
$\lambda_{2}$	variable for intermeeting time distribution of user and PoI
$E_{2}$	expected intermeeting time between user and PoI ($E_{2}=\frac{1}{\lambda_{2}}$)
$U_{i}$	utility of photo *i*
$P_{t_{i}}$	probability to finish delivering photo *i* to PoIs when $t_{i}$
$P_{r_{i}}$	probability to finish delivering the undelivered photo *i*
	when the remaining time is $r_{i}$
$P_{i}$	probability to finish delivering photo *i*
$D_{t_{i}}$	expected delivery delay for photo *i* after elapsed time $t_{i}$
$D_{r_{i}}$	expected delivery delay for photo *i* within remaining time $r_{i}$
$D_{i}$	delivery delay for photo *i*

**Table 2:** Simulation Parameters

Parameter	Random-Waypoint	Traces		
		Roma	Epfl	Geolife
Simulation Time	800,850,900,950,1000			
TTL	600∼800	700∼900		400∼600
Time Unit (s)	1	15	30	5
Number of PoIs	10	11	12	12
PoI Radius (m)	300	200	80	300
User Number	100	158	368	727
Connection Range	30	10	10	150
Storage Space	3∼7	5∼9	3∼7	8∼12
Photo Interval	1,2,3,4,5			
